# Environmental Suitability of the Sicilian Pond Turtle (*Emys trinacris*): An Approach Based on Bioclimatic and Environmental Variables for the Conservation of Sicilian Endemism

**DOI:** 10.3390/ani15233473

**Published:** 2025-12-02

**Authors:** Mario Lo Valvo, Grazia Orecchio, Maria Chiara Barone, Valentina Virgilio, Francesco Paolo Faraone

**Affiliations:** 1 Dipartimento di Scienze e Tecnologie Biologiche, Chimiche e Framaceutiche, Via Archirafi 18, I-90123 Palermo, Italy; grazia.orecchio@community.unipa.it (G.O.); francescopaolo.faraone@unipa.it (F.P.F.); 2 Via Ciaculli 7/C, I-90124 Palermo, Italy; 3 Dipartimento di Scienze della Terra e del Mare, Via Archirafi 18, I-90123 Palermo, Italy; valentina.virgilio@unipa.it

**Keywords:** Emydidae, Sicily, Maxent, endemic species

## Abstract

*Emys trinacris*, the Sicilian pond turtle, is a species endemic to the island of Sicily. Despite its global and Italian distribution aligning, *E. trinacris* is classified as “Data Deficient” by the IUCN Red List, but “Endangered” on the Italian Red List, due to threats from habitat destruction, pollution, invasive species, and the illegal pet trade. To aid conservation efforts, understanding the suitability of the species’ habitat is essential. This study aims to create a habitat suitability map by incorporating bioclimatic variables but also environmental factors related to the species’ preference for wetland habitats. We employed the Maximum Entropy model (MaxEnt), based on 264 georeferenced presence points and 33 climatic, topographic, and habitat-related variables. Our model, with an Area Under the Curve of 0.947 and True Skill Statistic of 0.853, identified key predictors such as winter temperature and summer precipitation, with a notable dependence on wetland vegetation. The resulting suitability map highlights the central-southern regions of Sicily as critical areas for the species, with moderate to high suitability also present in the western coastal areas. However, the map shows a discrepancy between the wide distribution of presence records and the limited high-suitability area. This study also assessed the overlap of suitable habitats with existing Natura 2000 sites, showing satisfactory protection levels, though agricultural reservoirs remain unprotected. Active conservation strategies, including expanding protected areas and improving habitat connectivity, are crucial to ensuring the long-term survival of *E. trinacris* in Sicily.

## Simple Summary

*Emys trinacris*, the Sicilian pond turtle, is a species endemic to the island of Sicily, threatened by habitat destruction, pollution, invasive species, and the illegal pet trade. This study aims to create a habitat suitability map by incorporating bioclimatic variables but also environmental factors related to the species’ preference for wetland habitats. We employed the Maximum Entropy model (MaxEnt). Our model identified key predictors such as winter temperature and summer precipitation, with a notable dependence on wetland vegetation. This study also assessed the overlap of suitable habitats with existing Natura 2000 sites, showing satisfactory protection levels.

## 1. Introduction

Until a few years ago, the population of pond turtles living in Sicily was considered a subspecies of the widespread (chorotype European-Mediterranean) European pond turtle, *Emys orbicularis* (Linnaeus, 1758). Later, based on phenotypic and genetic characters [1,2,3], this population was classified as belonging to a new species of freshwater turtle, *Emys trinacris* Fritz, Fattizzo, Guicking, Tripepi, Pennisi, Lenk, Joger, and Wink, 2005, commonly known as the Sicilian pond turtle and endemic to the island of Sicily, the largest island in the Mediterranean Sea. Although its specific rank has been subsequently debated [4], *E. trinacris* is still considered a valid species by several authors based on some experimental evidence [5].

*Emys trinacris* inhabits wetlands and slow-moving bodies of water (e.g., lagoons, deltas, inland waters, and mountain lakes) with soft bottoms and abundant aquatic vegetation, especially along the banks, from sea level up to 1036 m elevation [6]. Its distribution in Sicily is widespread but highly fragmented [7]. The assessment of the conservation status of this species is controversial. In fact, although the global and Italian range of this species are equivalent, according to the International Union for Conservation of Nature’s Global Red List, it is considered “Data Deficient” [8], while according to the Italian Red List, it is classified as Endangered (EN, A2c) [9,10]. The species appears to be mainly threatened by habitat destruction and fragmentation, pollution, the release of non-native species into the wild, and, in some cases, illegal capture for the pet trade [11]. *Emys trinacris* is listed in Annex II and Annex IV of the European Union Council Directive 92/43/EEC (“Habitats Directive”) and in Appendix II of the “Bern Convention on the Conservation of European Wildlife and Natural Habitats”.

Understanding the suitability of a species’ habitat, particularly when the species is considered threatened, as in the case of *E. trinacris*, is a critical first step in initiating targeted conservation actions. There are various ways to create a habitat suitability map, but the accuracy of the data and, especially, the selection of variables used to build the suitability map are crucial for obtaining useful results. An incorrect suitability map could be of limited value or, in the worst case, even harmful, especially for a species considered threatened.

Recently, Iannella et al. [12] produced models for *E. trinacris* based solely on current climatic conditions and inferred potential future changes in the distribution of the species under four global warming scenarios. Bioclimatic variables alone are not always sufficient to generate a suitable habitat map, especially for species whose presence is closely related to the availability of specific habitats, such as wetlands in the case of *E. trinacris*. In our study, we aimed to: (i) create a new environmental suitability map for *E. trinacris* based not only on bioclimatic variables but also on environmental variables; and (ii) assess the current ecological network of protected areas in relation to habitat suitability.

## 2. Materials and Methods

Numerous ecological models have been developed to predict the spatial distribution of species [13]. To define the suitability distribution model for *E. trinacris* in Sicily, we used the Maximum Entropy method, implemented through MaxEnt software (version 3.4.1). This method, often recognised as one of the best-performing modeling algorithms [14,15], unlike Generalised Linear Models and Generalised Additive Models, which require real absence data, uses pure machine learning techniques to model species distributions from presence-only records [14,16,17].

We modeled the ecological niches using georeferenced observations of *E. trinacris* from targeted sampling activities carried out by the authors, firsthand communications by colleagues and enthusiasts, and verified unpublished data collected from social media [e.g., *Fauna Siciliana* (https://www.facebook.com/groups/faunasiciliana/)] and naturalistic platforms [i-Naturalist (https://www.inaturalist.org/)]. The cell size used for the analysis was 1 km^2^, about double the maximum known distance for the species, which has low dispersal capacity [18,19], coinciding with the Universal Transverse Mercator (UTM) World Geodetic System 1984 grid.

We used 33 continuous variables related to climatic, topographic, and habitat features (Table 1). The climatic variables were extracted from the 19 Worldclim (ver. 2) dataset [20], with a 30 arc-second resolution (original resolution ≈ 1 km^2^ cell; average for 1970–2000).

To avoid multicollinearity between variable pairs, we assessed the degree of correlation by calculating the Pearson correlation coefficient. We then selected the most important factors [21] from pairs of variables with Pearson |r| > 0.85, as also noted in Iannella et al. [12]. Nine climatic variables were selected (BIO3, BIO4, BIO7, BIO11, BIO13, BIO16, BIO17, BIO18, and BIO19).

For a topographic variable, we used the average elevation (metres above sea level) derived from a 20 m resolution Digital Elevation Model. For habitat features, 23 habitat types (out of the 87 present in the Corine Biotopes maps of Sicily; scale 1:10,000) were selected, based on their presence within each 1 km^2^ UTM cell where *E. trinacris* records were located.

MaxEnt requires all environmental data to be placed in ASCII grids of the same resolution. Thus, each shapefile for individual habitat features was converted to raster format, where the value of surface area (in hectares) present in each 1 km^2^ cell was recorded. In this way, every habitat inside each cell was weighted. Models for *E. trinacris* were parameterised as follows: maximum iterations = 5000; beta multiplier = 2 to obtain smoother model responses [22]. The validation of the MaxEnt model was performed using Cross-Validation and the discrimination performance of the models was assessed through the Area Under the Curve (AUC) [16], Threshold (TH) and the True Skill Statistics (TSS) [23].

## 3. Results

**Figure 1:**
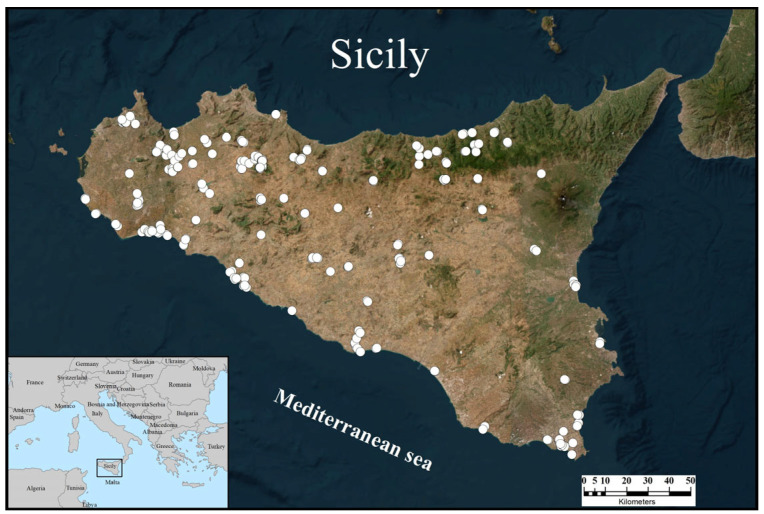
Distribution of the presence records (white dots) collected for *Emys trinacris* across Sicily.

A total of 264 presence points for *E. trinacris* were collected and georeferenced across the Sicilian territory (Figure 1).

The records were more abundant in the northern and southern parts of western Sicily, both in coastal areas and more inland regions. Presence records were also recorded in the eastern sector, though at a lower density. They are mainly concentrated in the coastal strip and scarcely present in the central-eastern part of the island. There are no records from the Peloritani Mountains (north-eastern Sicily) and all the surrounding islands.

**Figure 2:**
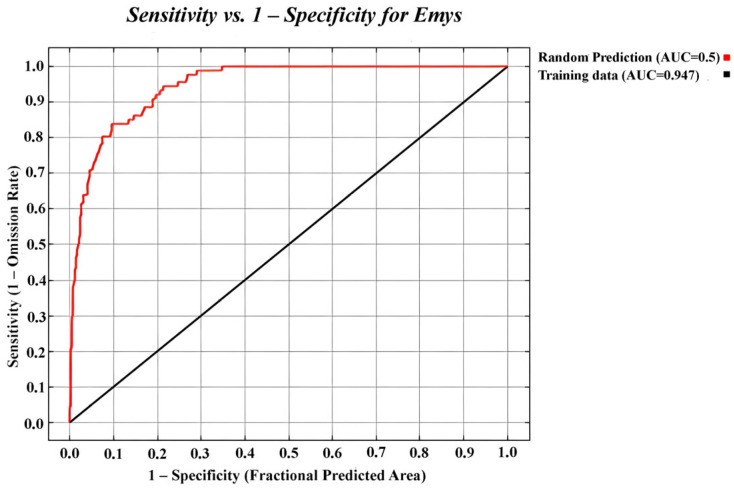
Receiver operating characteristic (ROC) curve for the sample data of *Emys trinacris*.

After eliminating autocorrelated observations, 170 records were selected for creating the suitability map. According to [24], the potential distribution model for *E. trinacris* showed very good overall performance with an AUC = 0.947 ± 0.03 (Figure 2), threshold = 0.13 and TSS = 0.853, indicating high predictive power.

Jackknife tests showed that the most important variables in the model were “BIO11 = Mean temperature of the coldest quarter” and “BIO17 = Precipitation of the driest quarter,” while the most suitable habitat was “41.21-Vegetation of wetland riverine and lacustrine habitats” (Table 1).

**Figure 3:**
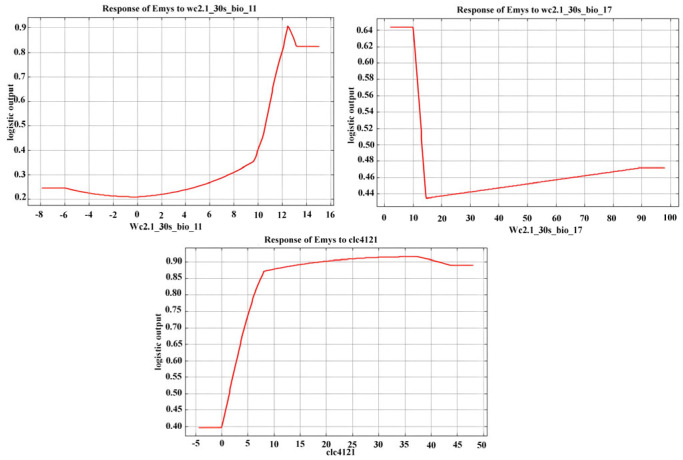
Marginal response curves obtained for the three highest contributing predictors of the distribution of *Emys trinacris*: BIO11 = Mean temperature of the coldest quarter; BIO17 = Precipitation of the driest quarter; 41.21-Vegetation of wetland riverine and lacustrine habitats.

Therefore, areas of Sicily where temperatures tend to be higher in winter (Figure 3) and with a higher probability of precipitation in summer (Figure 3) represent the most suitable habitats for the species, provided that wetland vegetation associated with the presence of water is well represented in these areas (Figure 3), which is necessary for a wetland species like *E. trinacris*.

**Figure 4:**
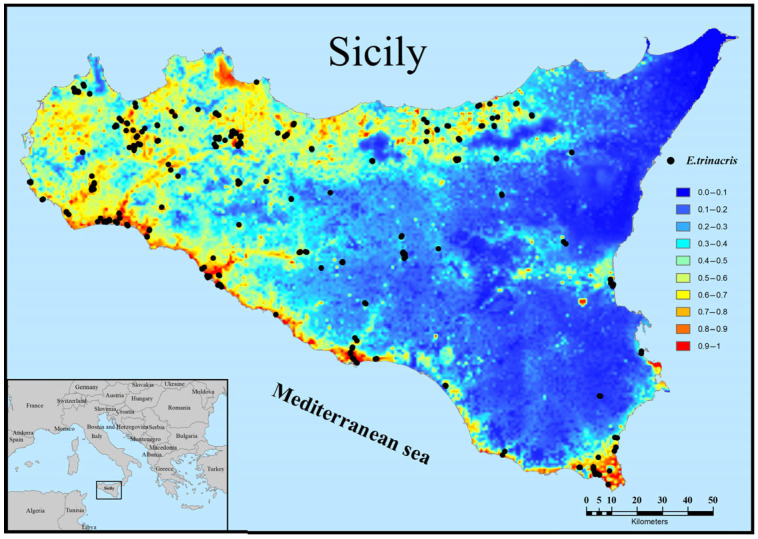
Habitat suitability and records for *Emys trinacris* in Sicily.

The predicted distribution of *E. trinacris* in Sicily is depicted in Figure 4, where the probability of presence ranges from 0 (unsuitable habitat) to 0.85 (optimal habitat). High and medium suitability areas are clustered in a limited region of central-southern Sicily, while the rest of the region shows low or no suitability. There is a notable correspondence between the largest high-suitability area and the majority of records. About 57% of the records selected for creating the suitability map fall within cells with a suitability value greater than 50%. Additionally, there are smaller suitable patches around the main area without records. Within the spatial extent of the observations used for the model, the average probability of presence is 0.31 (±0.27 standard deviation; minimum = 0.0004). The high variability and the near-zero minimum value reflect the species’ wide distribution, which spans from areas of highly optimal predicted suitability to sub-optimal habitats.

**Figure 5:**
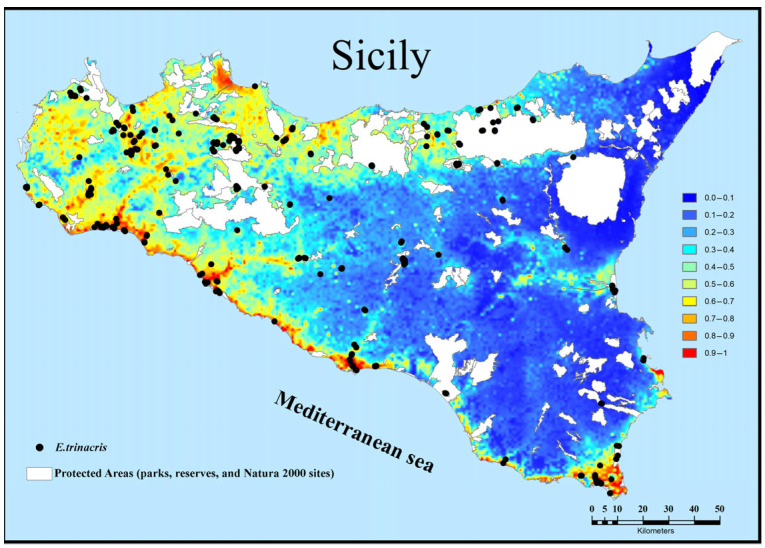
Overlay maps reporting of modelled suitability and presence records for *Emys trinacris* on current protected areas (parks, reserves, and Natura 2000 sites).

By overlaying our suitability map and observations with the map of protected areas (parks, reserves, and Natura 2000 sites), we find that there is a good level of protection for the species (about 60% of the records selected for creating the suitability map). However, a large, highly suitable area in western Sicily is characterised by a dense network of private agricultural ponds; the artificial and private nature of these important biotopes makes them difficult to amenable to conservation measures (Figure 5).

## 4. Discussion

Our dataset fills some gaps in previous assessments of the distribution of *E. trinacris* [8,25] by showing less fragmentation within the geographic range. On the other hand, our results confirm the absence or marginal presence in some areas of eastern Sicily such as the Peloritani mountains, Mount Etna, and the Hyblaean plateau. These are well-explored areas that have never provided evidence supporting the presence of the species. The Peloritani Mountains lack lakes or reservoirs (except for a few isolated artificial and mostly recreative ponds), and most of the watercourses are seasonal, apparently lacking suitable habitats.

An apparent inconsistency exists between the widespread presence records, particularly in northern and western Sicily, and the limited, clustered high-suitability area predicted by the MaxEnt model (Figure 4). While the records confirm the species’ existence across a wide geographic range, the model identifies only the small region in central-southern Sicily as combining the most optimal conditions—specifically, the combination of higher winter temperatures (BIO11), higher summer precipitation (BIO17), and high density of wetland vegetation (CLC 41.21). The records falling outside this core (approximately 43% of the data) likely represent populations persisting in sub-optimal or marginal habitats. This highlights the difference between the fundamental niche (modeled high suitability) and the realized niche (observed presence). This wide distribution, therefore, is responsible for the high dispersion observed in the statistics (average prob-ability of presence of 0.31 ± 0.27 and a minimum of 0.0004 within the area covered by the presence records). MaxEnt may not fully capture the influence of finer-scale factors, such as small, private agricultural ponds in western Sicily, that allow for persistence but are not dominant in the 1 km^2^ resolution environmental variables.

The first suitability map for *E. trinacris* was provided by Iannella et al. [12] through an Ensemble Modelling process based on presence and pseudo-absence data. This suitability map is less detailed than the one we obtained and only partially overlaps with it in the western area in terms of suitability and the northeastern portion of the island where the species is absent. However, it differs significantly in the central and southeastern portions of the island.

The contribution of the variables to the suitability model also differs. Iannella et al. [12] found that the evaluation of the contribution of each predictor in their model highlighted a clear predominance of bioclimatic variables related to precipitation, particularly the amount of precipitation in the wettest quarter (BIO16), the coldest quarter (BIO19), and the warmest quarter (BIO18), which contributed 31.2%, 23.0%, and 14.6% of the total contribution, respectively. In contrast, our model revealed that suitability is positively correlated with two climatic variables, (1) the mean temperature of the coldest quarter (BIO11), which, in various areas of Sicily, allows the species to remain active during part of the winter [18], and (2) the precipitation of the driest quarter (BIO17), which were found to be the best predictors.

A higher mean temperature in the coldest quarter could allow for a shorter or shallower brumation during winter. This prolongation of the active period might favor energy accumulation in an insular environment subject to summer water stress. The positive correlation with BIO17 (Precipitation of the Driest Quarter) could ensure the persistence of water bodies and the integrity of hygrophilous vegetation, mitigating the risk of desiccation during this period, particularly since a greater probability of summer precipitation is critical in an insular Mediterranean context where summer is the main period of water stress.

The differences between models may be attributed to several factors. Iannella et al. [12] used a database of 39 presence points (compared to 264 in our study), over half of which (n = 21; 54%) were imprecise or incorrect. In fact, 14 of the coordinates used by these authors and reported in Vamberger et al. [3] correspond to urban centres in towns where the species is cited but do not correspond to the actual locations where the species was observed. This resulted in the climate data used corresponding to urban environments rather than the true locations of the species. Furthermore, Iannella et al. [12] did not account for the presence of wetland environments, which are necessary for the survival of an aquatic turtle species.

Regarding the ecological network for this species, Iannella et al. [12] claimed that more than 50% of their observations did not fall within protected areas. But this percentage may be influenced by the fact that a third of the coordinates they used were within urban centres, which are typically not included in protected areas.

The environmental suitability map for *E. trinacris* in Sicily represents a crucial step in identifying the most critical areas for the species’ conservation. Our suitability model provided a detailed view of the most suitable areas for the species, highlighting the importance of fluvial and lacustrine wetland environments. However, in the context of rapid climate change, the stability of these areas could be threatened by changes in temperature and precipitation patterns. Rising winter temperatures and variability in precipitation could lead to the loss of suitable habitats, with potentially negative effects on *E. trinacris*, which depends on wetland environments for survival [8].

The loss or further reduction of suitable habitat area increases the risk of population fragmentation. These adversities could exacerbate existing threats, such as habitat destruction and the invasion of non-native species, worsening the species’ conservation status. As observed in other research on endemic and threatened species, habitat loss and changes in climate patterns are among the primary long-term threats to biodiversity [26]. It is therefore essential to continuously monitor environmental dynamics and implement targeted protection measures, such as the expansion of protected areas and active habitat management, to ensure the survival of *E. trinacris* in Sicily, especially in the context of ongoing climate change.

## 5. Conclusions

Climate change and alterations in temperature and precipitation patterns directly affect species distribution, as shown in predictive models applied to numerous other threatened species [21,27]. The inclusion of climatic variables in environmental suitability models and their continuous update are crucial to predict the future impacts of climate change and adapt conservation strategies. Furthermore, the importance of creating ecological corridors and ensuring connectivity between protected areas becomes even more relevant, as the ability of *E. trinacris* to move between suitable habitats could be crucial to facing future environmental challenges. Ultimately, the protection and management of *E. trinacris* should consider not only the conservation of natural habitats but also the global dynamics of climate change that affect the ecological balance of the region.

## Figures and Tables

**Table 1:** Estimates of the relative contributions of the climatic, topographic, and habitat variables to the Maxent model.

Variable	Percent Contribution	Permutation Importance
bio_11	30.5	30.5
Clc 41.21	10.7	1.6
bio_17	8.2	0.1

## Data Availability

Non-public data for privacy; contact the authors.
